# Serglycin in Quiescent and Proliferating Primary Endothelial Cells

**DOI:** 10.1371/journal.pone.0145584

**Published:** 2015-12-22

**Authors:** Trine M. Reine, Tram T. Vuong, Arkady Rutkovskiy, Astri J. Meen, Jarle Vaage, Trond G. Jenssen, Svein O. Kolset

**Affiliations:** 1 Department of Nutrition, Institute of Basic Medical Sciences, University of Oslo, Box 1046, Blindern, 0316 Oslo, Norway; 2 Department of Physiology, Institute of Basic Medical Sciences, University of Oslo, Oslo, Norway; 3 Department of Emergency and Intensive Care, Institute of Clinical Medicine, University of Oslo, Oslo, Norway; 4 Section of Renal Diseases, Department of Organ Transplantation, Oslo University Hospital–Rikshospitalet, Oslo, Norway; 5 Metabolic and Renal Research Group, UiT The Arctic University of Norway, Tromsø, Norway; Cedars-Sinai Medical Center, UNITED STATES

## Abstract

Proteoglycans are fundamental components of the endothelial barrier, but the functions of the proteoglycan serglycin in endothelium are less described. Our aim was to describe the roles of serglycin in processes relevant for endothelial dysfunction. Primary human umbilical vein endothelial cells (HUVEC) were cultured *in vitro* and the expression of proteoglycans was investigated. Dense cell cultures representing the quiescent endothelium coating the vasculature was compared to sparse activated cell cultures, relevant for diabetes, cancer and cardiovascular disease. Secretion of ^35^S- proteoglycans increased in sparse cultures, and we showed that serglycin is a major component of the cell-density sensitive proteoglycan population. In contrast to the other proteoglycans, serglycin expression and secretion was higher in proliferating compared to quiescent HUVEC. RNAi silencing of serglycin inhibited proliferation and wound healing, and serglycin expression and secretion was augmented by hypoxia, mechanical strain and IL-1β induced inflammation. Notably, the secretion of the angiogenic chemokine CCL2 resulting from IL-1β activation, was increased in serglycin knockdown cells, while angiopoietin was not affected. Both serglycin and CCL2 were secreted predominantly to the apical side of polarized HUVEC, and serglycin and CCL2 co-localized both in perinuclear areas and in vesicles. These results suggest functions for serglycin in endothelial cells trough interactions with partner molecules, in biological processes with relevance for diabetic complications, cardiovascular disease and cancer development.

## Introduction

The endothelium forms the inner lining of the vasculature and have important barrier functions, involving extracellular matrix components located both in the basolateral basement membrane and in the glycocalyx exposed on the apical side of the cells facing the circulation [1]. Proteoglycans are important components of both of these matrices [2, 3]. Proteoglycans are proteins substituted with unique sugar chains; glycosaminoglycans; having the ability to interact with partners molecules including chemokines, growth factors and proteases. The endothelium is a metabolically active organ with impact on a range of key processes including growth, vasomotor activity, lipid metabolism and coagulation [4], as well as inflammation and extravasation of immune cells. Endothelial dysfunction is receiving increasing attention in relation to diabetes [5], cardiovascular disease [6] and cancer [7]. Inflammation is an important aspect of diabetes, cancer and cardiovascular disease where endothelial cells may play an active role through their synthesis of inflammatory molecules such as cytokines, chemokines, proteoglycans and other secretory products [8, 9]. IL.1β has emerged as an important factor in the pathogenesis of type 2 diabetes [10]

One important consequence of endothelial dysfunction is changes in processes involving injury and repair. Inflammation, angiogenesis and proliferation are all players in the complex process of wound healing, and must be tightly regulated. Dysregulation of these processes potentially result in diabetic complications [11]. Abnormal angiogenesis is seen in diabetes [12], and anti-angiogenic approaches using e.g. anti-VEGF treatment is currently being tested in treatment of retinopathy [13]. Angiogenesis is also a process with high relevance to tumor biology and metastasis [14] and inflammatory mediators are essential components also of the tumor microenvironment [15].

Primary human umbilical vein endothelial cells (HUVEC) have been widely used in studies on endothelial cells, also in relation to diabetes [16–18]. Exposure to inflammatory conditions *in vitro* affects both pro-adhesive properties [19] and the structure of heparan sulfate expressed and released by these cells [20]. In polarized HUVEC serglycin is a major proteoglycan and it is secreted to the apical medium. Activation with IL-1β increased the secretion of serglycin which co-localized with the chemokine CXCL1 (GRO-α) in type 2 granula [21]. This suggests interplay between serglycin and inflammatory mediators. Furthermore, due to high expression in hematopoietic cells, serglycin is primarily regarded as a proteoglycan linked to inflammation [22], and it can be hypothesized that similar immunological functions of serglycin may be relevant for endothelial cells. Also, the involvement of serglycin in inflammation, proliferation and angiogenesis may implicate serglycin in several cancers [23–26].

We here study the functions and mechanisms of the proteoglycan serglycin in primary endothelial cells. We hypothesized that serglycin is involved in processes relevant for endothelial dysfunction through interactions with partner molecules through its glycosaminoglycan side chains. Such processes include proliferation and angiogenesis such as in wound healing, and inflammation. Serglycin expression was studied in proliferating cells, relevant for studies of angiogenesis and inflammation, and confluent cultures, representing the quiescent vascular endothelium lining our blood vessels.

## Experimental procedures

### Cell culture

HUVEC were isolated from umbilical cords as described [27]. Written informed consent was obtained from each donor, and the use was also approved by *The Regional Committees for Medical and Health Research Ethics* (REK). Cells were cultured at 37°C and 5.0% CO_2_ in MCDB 131 medium (Sigma) containing 5 mM glucose and supplemented with 7% heat-inactivated fetal calf serum (FCS, Sigma), basic fibroblast growth factor (bFGF, 1 ng/ml, R&D), hydrocortisone (1 μg/ml, Sigma), epidermal growth factor (EGF, 10 ng/ml, R&D), gentamicine (50 μg/ml, GIBCO Invitrogen) and fungizone (250 ng/ml, GIBCO Invitrogen). The medium was replaced three times a week and cells were used for experiments within three passages. The purity of the endothelial cell cultures was verified by microscopic observations of each culture as well as regular staining for the endothelial cell marker von Willebrand factor (vWF). For experiments, cells were seeded in wells or chamber slides at different cell densities. Sparse cell cultures were obtained by seeding approximately 15 000 cells/cm^2^ and dense cell cultures were obtained by seeding approximately 100 000 cells/cm^2^. The contact-inhibition of dense cell cultures promote quiescence [28]. For all experiments the cells were allowed to adhere, and incubated for up to 24 hours before the experiment was initiated. Incubations with IL-1β (R&D systems) were performed for 24 hours using a concentration of 0.5 ng/ml; determined by dose-response and time-response experiments (not shown).

### Protein determination

Protein content of cell lysates was determined using Uptima BC Assay protein quantization kit (BioRad) according to the manufacturer’s protocol.

### Cytotoxicity assay

The possible cytotoxic effect of cellular density as well as IL-1β exposure was investigated by measurement of LDH activity released from damaged cells, using the Cytotoxicity Detection Kit (Cat.no 11 644 793 001, Roche) according to the manufacturer’s instructions. In brief, conditioned media from cells cultured at different cellular densities, alternatively with or without IL-1β, was harvested; assay medium was added and incubated at 37°C for 30 min before recording the absorbance at 490 nm. Conditioned media from Triton X-100 treated cells were included as positive control, and non-conditioned medium was included as negative control.

### Proliferation assays

Cellular growth rate of sparse versus dense cell cultures was assessed using the MTS assay, performed according to the manufacturer’s descriptions (CellTiter 96 Aqueous One Solution Cell Proliferation; G3582; Promega). In short, cells were seeded in triplicates in varying densities in 96-wells plates in 100 μl culture medium. Twenty μl of MTS reagent was added directly to the wells and incubated for two hours. The quantity of farmazan bioreduced from MTS by the cells was then measured by recording the absorbance at 490 nm. An increased absorbance is a measure of increased proliferation rate.

The effect of knockdown of serglycin expression on proliferation was determined using the CyQuant Direct Cell Proliferation Assay Kit (C35011, Molecular Probes) according to the manufacturer’s descriptions. Briefly; siSRGN or siScramble transfected cells were seeded at 10 000 cells per well in triplicates on black plate clear bottom 96-wells plates (3603, Corning) and cultured overnight in 100 μl culture medium. The proliferative capacity of subconfluent cultures was quantified after incubation with Detection Reagent and subsequent fluorescence detection at 480/535 nm. An increase in fluorescence intensity is a measure of DNA content and thus an increased proliferation rate.

### Metabolic labeling

HUVEC cultures of the desired density were metabolically labeled with 0.1 mCi/ml ^35^S-sulfate (Hartmann Analytic) in RPMI-1640 sulfate free medium (GIBCO Invitrogen) added 5 mM L-glutamine (Sigma) and with FCS reduced from 7 to 2% to increase labeling efficiency. After labeling for 24 hours, the culture medium was collected. The cells were washed in PBS and harvested in either lysis buffer (4.0 M guanidine-HCl, 0.1 M acetate buffer pH 6.5, 2% Triton X-100) or RIPA buffer (50 mM Tris-HCl pH 7.4, 150 mM NaCl, 1% Triton X-100, 1% SDS, 1% Na-deoxycholate, 10 mM EDTA, 10 mM Na_4_P_2_O_7_ and phosphatase inhibitor tablet freshly added). In order to remove unincorporated ^35^S-sulfate, samples were subjected to Sephadex G50 fine (GE Healthcare) gel chromatography in buffer (0.05 M Tris-HCl, 0.05 M NaCl, pH 8). The ^35^S -macromolecules were eluted in the void volume, while smaller molecules remained associated with the column. The amount of ^35^S-sulfate incorporated in newly synthesized ^35^S -macromolecules was determined by scintillation counting in triplicates. ^35^S-macromolecules in HUVEC are almost exclusively comprised of PGs [16]. Protein content of cell fractions was determined in RIPA-lysates prior to G50 fine gel chromatography or in guanidine-lysates after changing the buffer on the G50 fine column.

### SDS-PAGE

Conditioned medium fractions containing equal amounts of radiolabeled material within each experiment, approximately 10,000–15,000 cpm, were concentrated using vacuum centrifugation and treated with nitrous acid (HNO_2_), chondroitin ABC lyase (cABC) or trypsin, or left untreated, before subjected to SDS-PAGE at 110V for two hours. Heparan sulfate was depolymerized by HNO_2_ deamination at pH 1.5, cleaving the polysaccharide at N-sulfated glucosamine units as described [29]. Chondroitin sulfate (CS) and dermatan sulfate (DS) was degraded by incubation at 37°C overnight with 0.01–0.02 units of cABC (E.C.4.2.2.4, Sigma C2905 or Seikagaku 100330) in 0.05 M Tris-HCl pH 8.0 containing 0.05 M sodium acetate and 0.02% BSA. For identification of the protease-resistant proteoglycan serglycin, samples were treated with 5 μl 0.25% trypsin-EDTA (T4049, Sigma) at 37°C overnight, before inactivation with an equal volume of 2.5% soy bean trypsin inhibitor (SBTI, Sigma). Prior to loading, the samples were incubated at room temperature for 15 minutes in Laemmli buffer. After completed electrophoresis the 4–20% gradient-gels were treated with fixing solution (isopropanol 25%, glacial acetic acid 10%) and Amplify (Amersham). The gels were dried and subjected to fluorography using Amersham Hyperfilm™ ECL for approximately four days at -80°C.

### Western blotting

HUVEC were established at different densities in MCDB-131 medium as described, but with serum reduced to 2%. After 24 hours the medium was collected and cell debris removed by centrifugation. For protein measurements, cells were washed in cold PBS and lysed in RIPA buffer. The sample volumes from the conditioned medium were adjusted to the protein content of the corresponding cell fractions. Laemmli buffer was added and conditioned medium samples subjected to SDS-PAGE on 4–20% gradient gels and electroblotted onto PVDF membranes (Millipore) using the Criterion™gel system (BioRad). Primary antibodies monoclonal mouse anti-human perlecan (MAB1948, Millipore, 1:500) 1:20,000) and monoclonal goat anti-human biglycan (NB100-55407, Acris, 1:5,000) were used. The secondary antibodies used were HRP-linked sheep anti-mouse IgG (NAV931, Amersham, 1:500) and HRP-linked rabbit anti-goat IgG (HAF017, R&D, 1:50,000). The membranes were developed using ECL Western Blotting Detection Reagents (GE Healthcare) and finally exposed to films (Amersham Hyperfilm™ ECL). The bands were quantified using the densitometric quantification software *Image J*. All antibodies used are listed in Table 1.

**Table 1:** Antibodies.

Antibody		supplier	dilution	application
Monoclonal Mouse anti-human perlecan	Primary	MAB1948, Millipore	1:500	western
Monoclonal Goat anti-human biglycan	primary	NB100-55407, Acris	1:5000	western
Monoclonal Mouse anti-human serglycin	Primary	H00005552-H03, AbNova	1:500	ip
Polyclonal Rabbit anti-human serglycin	Primary	HPA000759, Atlas Antibodies	1:100	ip
Polyclonal Rabbit anti-human versican	primary	Gift from Dieter Zimmerman, Zurich	1:100	ip
Monoclonal Mouse anti IL-33	primary	Clone Nessy-1, Enzo Life sciences	1:200	ICC
Polyclonal Rabbit anti-human serglycin	primary	Gift from Niels Borregaard, Denmark	1 μg/ml	ICC
Monoclonal Mouse anti-KDEL	primary	10C3, Millipore	1:200	ICC
Monoclonal Mouse anti-GM130	primary	BD Biosciences	1:250	ICC
Monoclonal Mouse anti-human vWF	primary	Dako Cytomation	1:1,400	ICC
Monoclonal Mouse anti-human CCL2	primary	ABIN969505, Antibodies Online	1:400	ICC
Rabbit-anti Hemocyanin (KLH)	primary	H0892, Sigma	1 μg/ml	ICC
Biotinylated horse anti-mouse IgG	secondary	Vector labs	1:200	ICC
HRP-linked rabbit anti-goat IgG	secondary	HAF017, R&D	1:50 000	western
HRP-linked anti-rabbit IgG	secondary	NA934, GE Healthcare	Cons matched	western
HRP-linked sheep anti-mouse IgG	secondary	NAV931, Amersham	1:5000	western
Alexa Fluor 488 conjugated goat anti-rabbit IgG	secondary	Invitrogen	1:600	ICC
Alexa Fluor 488 conjugated goat anti-mouse IgG	secondary	Invitrogen	1:600	ICC
Alexa Fluor 546 conjugated goat anti-mouse IgG	secondary	Invitrogen	1:600	ICC
Alexa Fluor 546 conjugated goat anti-rabbit IgG	secondary	Invitrogen	1:600	ICC
Streptavidin Cy3-conjugate	tertiary	016-160-084, Jackson	1:1000	ICC

### Imunoprecipitation

HUVEC were incubated with ^35^S-sulfate and cell and medium fractions recovered as described above. The volumes of the conditioned media were adjusted to the protein content of the corresponding cell fractions, and subjected to G50-fine gel chromatography. The samples were divided in two, one was left untreated and one was subjected to cABC treatment. Conditioned medium of equal protein concentration (related to their corresponding cell lysates) and containing approximately 40,000 cpm were incubated over night with agitation at 4°C with monoclonal mouse anti human serglycin (H00005552-H03, Abnova, 1:500) or polyclonal rabbit anti-human serglycin (HPA000759, Atlas Antibodies, 1:100) or polyclonal rabbit anti-versican (a kind gift from Dieter Zimmermann, University Hospital Zurich). In initial experiments medium from the monocytic cell line THP-1 was included as positive control for serglycin and unconditioned cell culture medium or concentration matched irrelevant IgG control (Mouse Gamma Globulin, Jackson Immuno Research, 015-000-002) were included as negative control. Further, 30 μl Protein A/G solution (sc-3003, Santa Cruz) was added and the incubation was continued for two hours. The samples were then centrifuged and washed three times in 0.05 M Tris-HCl with 0.15 M NaCl, 0.05% Triton X-100 and 1% BSA, followed by one wash in PBS. Material bound to Protein A/G was finally released by boiling for five minutes in Laemmli sample buffer, centrifuged and loaded on to 4–20% SDS-PAGE. Molecular weight protein markers (BioRad) were run on all gels for size determination. After electrophoresis the gels were dried and subj ected to autoradiography. The intensity of the bands were quantified using the densitometric quantification software *Image J*.

### ELISA assays

Conditioned medium was collected and centrifuged to remove cell debris, and shedded syndecan-4 as well as secreted CCL3, VEGF and CCL2 were detected according to the manufacturer’s instructions using the following ELISA kits: Human Syndecan-4 Assay Kit (JP27188, IBL), Human CCL3/MIP-1 alpha DuoSet (DY270-05, R&D), Human VEGF Quantikine ELISA Kit (DVE00, R&D) and Human CCL2/MCP-1 DuoSet, (DY279-05, R&D).

### Gene expression analysis

Total RNA was isolated from cultured HUVEC using the E.Z.N.A. Total RNA kit 1 (R6834-02, Omega Bio-Tek) according to the manufacturer’s instructions. RNA quantity measurements were performed using the ND1000 Spectrophotometer (Saveen Werner) and RNA was stored at -80°C until further analysis. A quantity of 250 ng RNA was reverse transcribed in a total volume of 20 μl using the “High capacity RNA-to-cDNA kit” (cat no 4387406, Applied Biosystems). Quantitative Real-time PCR (qRT-PCR) was performed on an ABI Prism 7900HT (Applied Biosystems) using Taq Man Gene Expression Master Mix (4369016, Applied Biosystems) and predesigned TaqMan Gene Expression Assays as listed in Table 2. 60S ribosomal protein L30 (*RPL30)* was used as endogenous control. The Ct cutoff value was set to 40 cycles, and the relative mRNA level for each transcript was calculated by the ∆∆Ct method [30]. Briefly, the cycle threshold (Ct) values for each gene was normalized against the Ct values of the housekeeping gene (= ∆Ct). For comparison of gene expression in treated versus control cells, ∆∆Ct was calculated as ∆Ct in treated cells subtracted the ∆Ct for control cells. The fold change in mRNA expression was calculated as 2^-∆∆Ct^.

**Table 2:** TaqMan gene expression assays used in qRT-PCR analysis.

Gene	Protein	Assay ID
*HSPG2*	Perlecan	Hs00194179_m1
*SRGN*	Serglycin	Hs01004159_m1
*SDC4*	Syndecan-4	Hs00161617_m1
*BGN*	Biglycan	Hs00156076_m1
*VCAN*	Versican	Hs00171642_m1
*IL-33*	Interleukin-33	Hs00369211_m1
*MKI67*	Marker of proliferation Ki-67	Hs01032443_m1
*ANG2*	Angiopoietin-2	Hs01048042_m1
*CCL2*	CCL2	Hs00234140_m1
*RPL30*	60S ribosomal protein L30	Hs00265497_m1

### Immunocytochemistry

Sparse and dense cultures of HUVEC were grown for 24 hours on Lab-Tek chamber slides (Nalge Nunc International) coated with 1% (w/v) gelatin from porcine skin. For IL-33 staining, the slides were washed in PBS and fixed in ice cold methanol for 10 min and stored at 4°C. The fixed cells were labeled with mouse monoclonal anti-human IL-33 (clone Nessy-1; Enzo Life Sciences, 1:200) at 4°C overnight in a dark humidity chamber, followed by secondary biotinylated horse anti-mouse IgG (Vector labs, 1:200) for 1.5 hours at room temperature, and finally tertiary antibody streptavidin Cy3-conjugate (Cat.no. 016-160-084, Jackson, 1:1000) for one hour. For staining with the other antibodies, the slides were submerged three times in PBS, fixed in 4% paraformaldehyde for 10 min, rinsed in PBS for 10 minutes and finally dipped in milli-Q water. The slides were dried and stored at 4°C until staining. The fixed cells were labeled with affinity purified rabbit anti-human serglycin (1 μg/ml, kindly provided by Professor Niels Borregaard, University of Copenhagen), monoclonal mouse anti-KDEL/GRP78 BiP (10C3, Millipore, 1:200), monoclonal mouse anti-GM130 (BD Biosciences, 1:250), monoclonal mouse anti-human vWF (Dako Cytomation, 1:1,400) or monoclonal mouse anti human CCL2 (Antibodies online, 1:400) antibodies, overnight at 4°C in a dark humidity chamber. All antibodies were diluted in PBS containing 1.25% BSA and 0.2% saponin for permeabilization. The slides were washed 10 minutes in PBS and incubated with Alexa Fluor 488 conjugated goat anti-rabbit IgG (Invitrogen, 1:600) and/or Alexa Fluor 546 conjugated goat anti-mouse IgG (InVitrogen, 1:600) for 90 minutes at room temperature. Negative controls were prepared by substituting diluting buffer for primary antibody or by using an irrelevant concentration matched antibody (Rabbit-anti Hemocyanin (KLH), H0892, Sigma) as negative control for serglycin. All antibodies used are listed in Table 1. After staining, the slides were washed for 10 minutes in PBS, dipped in milli-Q water, air dried, and mounted using SlowFade Gold antifade reagent with DAPI (Invitrogen). Fixed cells were examined using confocal microscopy. This was acquired using an Olympus FluoView FV1000 (Olympus Corporation) with a plan apochromat 60x/1.35 oil objective. All images were taken as single sections in the z-plane. Triple-stained images were obtained by sequential scanning for each channel to eliminate the crosstalk of chromophores and to ensure reliable co-localization. Alternatively, cells were examined with a Zeiss LSM 700, confocal laser-scanning microscope (Zeiss Microlmaging GmbH), using the Zen software (Zen 2011 black edition/Zeizz) taken on 62x magnification. Z-stack reconstructions were made. Images were then processed using Adobe Photoshop and Adobe Illustrator CS4/CS5 or Adobe InDesign CS6.

### Application of cyclic stretch–FlexCell

HUVEC was cultured on flexible-bottomed culture plates and grown to confluence, before subjected to a 10% sinusoidal wave elongation at 1.0 Hz for 4 or 24 hours. As described, mRNA was obtained, and the effect of cyclic stretch on serglycin expression was determined by qRT-PCR.

### Culture of polarized cells

Polarized cells were obtained as described in Meen et. al. [21]. HUVEC were seeded on Costar Transwell clear polyester membrane inserts with a pore size of 0.4 (Sigma-Aldrich) in 12-well plates. The cells were seeded at a density of 1 × 10^5^ cells/cm^2^ with 1.5 ml of culture medium in the basolateral compartment and 0.5 ml in the apical compartment. Medium was changed every second day until the start of the experiment, when a tight monolayer had been established.

### Serglycin knockdown

The expression of human serglycin in HUVEC was reduced using SRGN siRNA (sc-44093, Santa Cruz) and the negative control siRNA (scramble, sc-37007, Santa Cruz) at 0.02 μM. HUVEC were reversly transfected with siPORT (AM4502, Ambion) in Opti-MEM (Invitrogen) at a cell density of 200 000 cells per ml (25 000 cells / cm^2^), allowed to adhere and incubated for 5 hours. Then, the transfection medium was replaced with MCDB growth medium for 19 hours, followed by a second 24 hours transfection in OptiMEM. After yet another 24 hours, the silencing efficiency was quantified by qRT-PCR and immunohistochemical stainings. The scramble controls did not differ from the untreated controls, indicating no effect on siRNA transfection per se.

### In Vitro Angiogenesis assays

The impact of serglycin on endothelial cell tube formation was investigated using the Cultrex^®^ *In Vitro Angiogenesis Assay Kit Tube Formation* (3470-096-K, Amsbio) according to the manufacturer’s instructions. In short, HUVEC transfected with siSRGN and siScramble control, as well as nontransfected controls, were seeded on the reduced growth factor basement membrane extract (BME) gel in triplicates on a 96 wells plate at 10 000 cells per well for 5 hours. This was done in growth medium containing growth factors and 7% serum. Sulforaphane which inhibits tube formation was included as a negative control, as well as cells cultured in medium without growth factors or serum. The cells were stained with 2 μM Calcein AM, allowing visualization of the tube formation using a fluorescence microscope. Images were recorded with a Leica DM IL inverted contrast microscope with a Leica DFC420 digital color camera, and submitted to the Wimasis online quantification service quantifying several parameters, including the number of loops, mean tube length and mean loop perimeter [31].

### Wound healing assay

Confluent, quiescent cultures of siSRGN or siScramble transfected cells, as well as nontransfected controls, were exposed to a scratch wound using a 200 μl sterile pipette tip. The rate of wound closure was visually monitored, and quantified by the mean change in wound width.

### Hypoxia

Six-well plates with attached HUVECs were placed in the hypoxia chamber (Cat.# 865-HYPO, Plas Labs) at 0.5% oxygen for 24 hours. Conditioned media was harvested and RNA was isolated immediately.

### Statistics

Comparative analysis of the data was carried out using the Student’s paired *t*-test. Data were presented as mean ± SEM of the indicated number (n) of experiments. *P*-values < 0.05 were considered to be statistically significant.

## Results

### Cellular density

**Fig 1:**
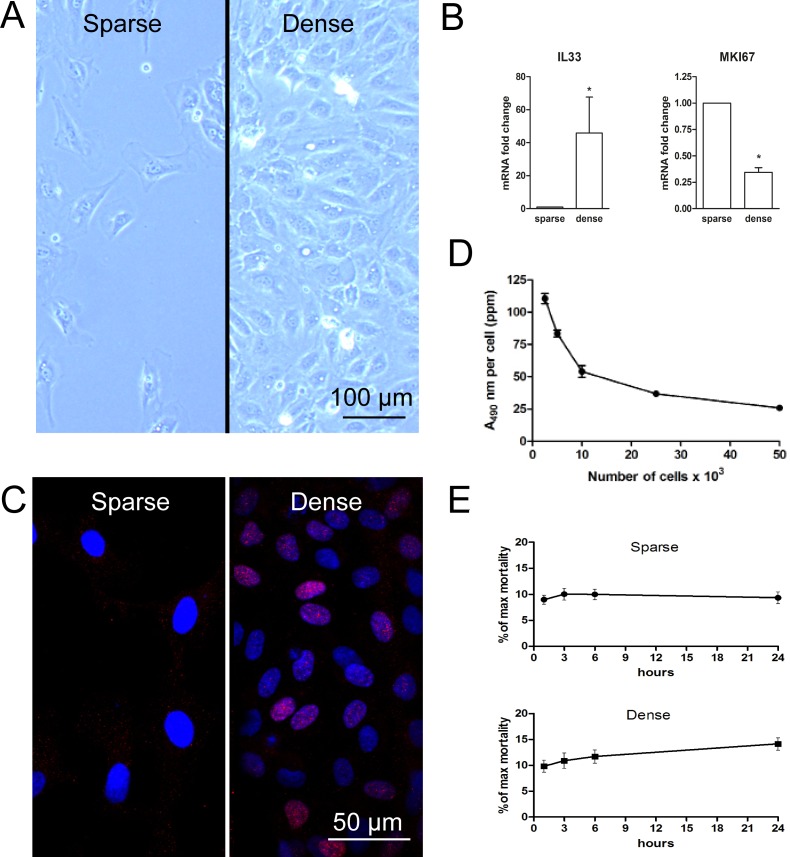
Cellular proliferative state. HUVEC were cultured in low (sparse) and high (dense) densities. (A) Illustration of the morphology of sparse (left panel) and dense (right panel) HUVEC. (B) Gene expression of quiescence marker IL33 and proliferation marker MKI67 in sparse relative to dense cell cultures determined by qRT-PCR in material from six donors. (C) Staining for IL-33 in dense culture (right panel) and sparse culture (left panel) visible as magenta, costained with DAPI nuclear staining visible as blue. The pictures were acquired using a confocal microscope at 60X magnification. (D) The proliferation rate was assessed by applying the MTS-assay on cell cultures of increasing densities from four donors. (E) Cell mortality rate was determined with the LDH-assay for 5 donors in both sparse (upper panel) and dense (lower panel) cell cultures. All data are presented as means with SEM, and statistical significant differences (*p* < 0.05) was tested with Students paired *t*-test and denoted by *. The scale bars indicates 100 and 50 μm respectively.

Sparse and dense cultures of primary endothelial cells could represent useful models for different *in vivo* situations. Dense cell cultures are contact-inhibited and non-proliferative, resembling the quiescent endothelium coating the vasculature. In contrast, sparse cell cultures are proliferating and can be used as an experimental model system with relevance in wound healing and neoplasia. The typical morphology of a sparse and a corresponding dense culture is shown in Fig 1A, illustrating the typical fibroblastic appearance of sparse proliferating cells and the cobblestone appearance of dense quiescent cells.

IL-33 in its mature cleaved form is a proinflammatory cytokine, while its full length precursor acts as a nuclear factor. In endothelial cells, nuclear IL-33 is expressed only in the quiescent state and is regarded as a marker for endothelial quiescence [32]. The Ki-67 (MKI67) protein, in contrast, is a cellular marker of proliferation [33]. To verify the proliferative status of our cell cultures, gene expression of these two markers was determined in sparse and dense cultures (Fig 1B). The increased expression of IL-33 and decreased expression of MKI67 confirmed the quiescent state of the dense cultures. Accordingly, the low IL-33 and high MKI67 expression in sparse cultures confirmed that these cells were in a proliferative state. In addition, both sparse and dense cultures were immunostained for IL-33 (Fig 1C). DAPI-stained nuclei are visible in blue, while IL-33 is seen as magenta stain. The nuclear presence of IL-33 in dense cultures only confirmed the quiescent state of dense cultures.

The effect of cellular density on cell proliferation and mortality was determined using the MTS-assay and the LDH-assay respectively. From Fig 1D it is evident that the proliferation of HUVEC is inversely correlated to cell density. To investigate the effect of cell density on viability, the LDH-assay was used. Fig 1E shows that LDH-leakage was not significantly increased over time, neither in sparse nor in dense cultures.

### Proteoglycan secretion

**Fig 2:**
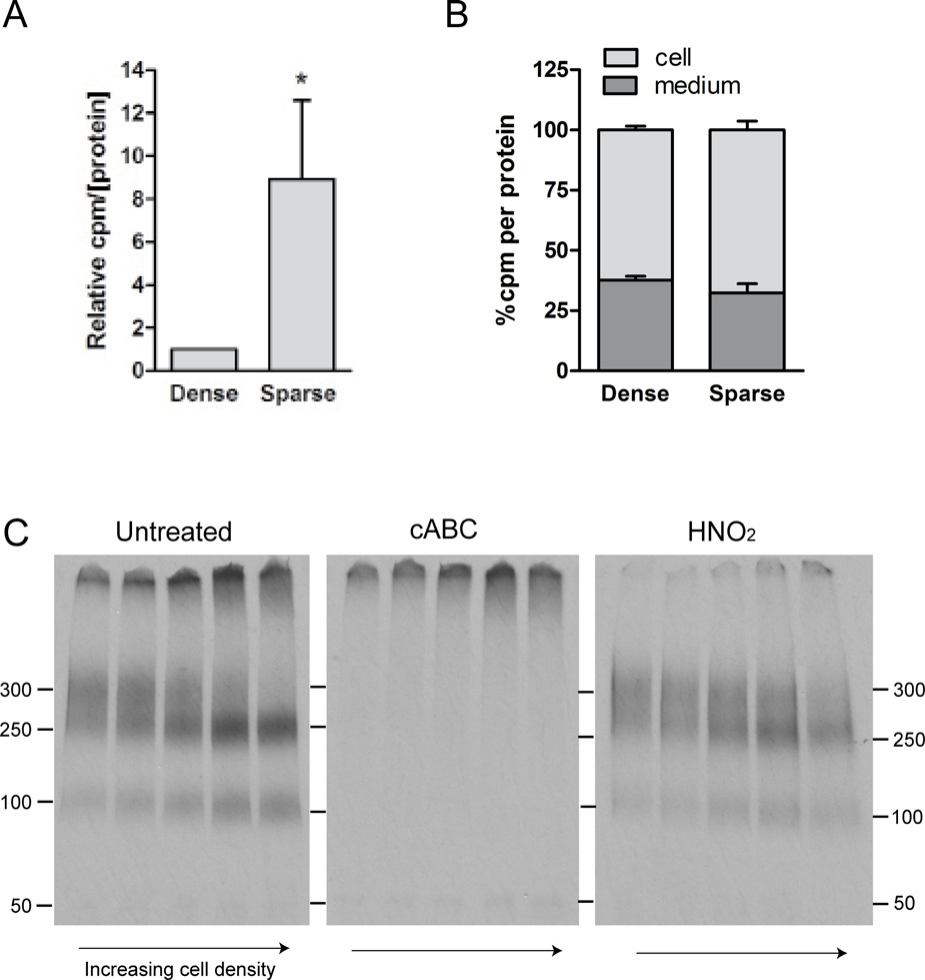
^35^S-proteoglycan secretion and cellular density. Sparse and dense cultures of HUVEC were metabolically labeled for 24 hours with ^35^S-sulfate, and ^35^S- macromolecules were recovered. (A) The amount of secreted *de novo* synthesized ^35^S-PGs from 6 donors was determined by scintillation counting and normalized to protein. The presented results are relative to the mean of the dense values. (B) The % distribution of ^35^S-PGs related to protein detected in cell lysate (cell) and in conditioned medium (medium). Mean values from both sparse and dense cultures originating from 5 donors are shown. Results are presented as mean with SEM denoted by vertical bars, and *p*-values * < 0.05 were taken as a significant difference between the sparse relative to dense cells using the Students paired *t*-test. (C) Secreted ^35^S-PGs were harvested from cell cultures of increasing densities, indicated by arrows. Equal amounts (cpm) were loaded in each lane and separated using SDS-PAGE. We here show one representative of 6 individual experiments. The migration positions of molecular mass markers are given in kDa. (D The intensity of the bands was quantified using *Image J* and mean ± SEM is presented.

A common feature of proteoglycans is their heavily sulfated glycosaminoglycan-chains, providing these molecules with the unique ability to bind to biologically active partner molecules based on electrostatic- or sequence-specific interactions. HUVEC were metabolically labeled with ^35^S-sulfate, and the effect of cellular density on proteoglycan expression *per se* was studied. ^35^S-proteoglycan secretion increased ~ 9 times (± 3.7) in sparse compared to dense cell cultures (Fig 2A). Cellular density had no significant effect on the distribution of ^35^S- proteoglycans between the medium and the cell fractions, the medium contained 67 and 62% of the total ^35^S- proteoglycans in sparse and dense cultures, respectively (Fig 2B).

The size of the secreted ^35^S- proteoglycans was analyzed by SDS-PAGE. To focus on the relative ratios of the respective ^35^S- proteoglycans, equal amounts (cpm) of material were loaded in each lane. The material was separated into three distinct ^35^S- proteoglycan populations; one at the top of the gel, a second at molecular weights ranging from approximately 250 to 300 kDa and a third at 100 kDa (Fig 2C, left panel). We identified the upper band as predominately heparan sulfate proteoglycans by its susceptibility to HNO_2_ depolymerization (right panel), while the 250–300 kDa and the 100 kDa bands were of the CS/DS type, as they were susceptible to cABC digestion (middle panel). This is in line with earlier observations [20, 21], and here we show that this is the case in both sparse and dense cell cultures. The most striking effect of cell density was observed for the high molecular weight CS/DS proteoglycan population. With increasing density there was an obvious decrease in the 300 kDa part and an increase in the 250 kDa part of this material.

**Fig 3:**
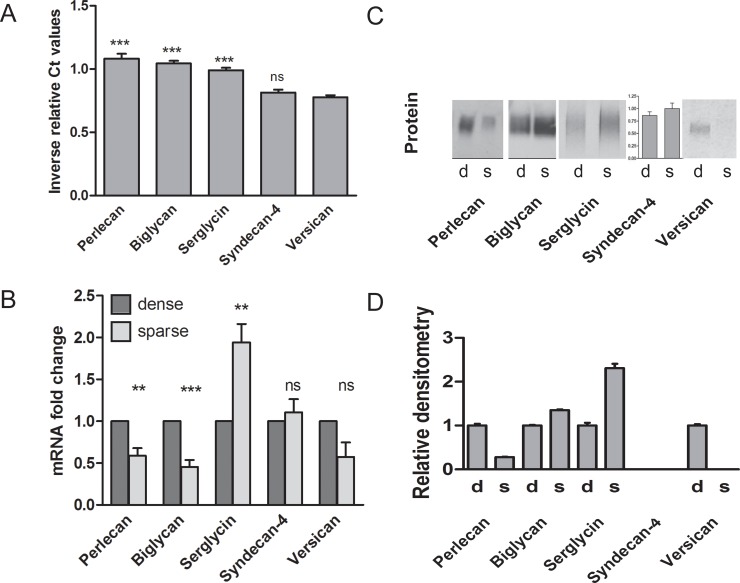
Major PGs expressed and secreted by HUVEC. (A) Gene expression levels of perlecan, biglycan, serglycin, syndecan-4 and versican in sparse cultures of HUVEC determined by qRT-PCR was expressed as the inverse of the Ct value relative to the Ct value of the endogenous control gene RPL30. The number of different primary cultures was 7 for all genes except for biglycan and versican resulting from 3 cultures. Differences relative to versican are tested for statistical significanse. (B) Gene expression of perlecan, biglycan, serglycin, syndecan-4 and versican in HUVEC cultured in low (sparse) compared to high (dense) cell densities was determined by qRT-PCR. Values are expressed as the fold change in sparse relative to dense cells. The number of primary cell culture donors was 7, 8, 8, 10 and 6 respectively. (C) The secretion of perlecan, biglycan, serglycin, and versican from dense (d) and sparse (s) HUVEC cultures was compared performing western blotting (perlecan and biglycan) or immunoprecipitation (serglycin and versican). The sample size was adjusted to the protein content of the corresponding cell lysate, and shown are representative results from four donors. Similarly, syndecan-4 shedded to the medium was measured by ELISA in sparse and dense cultures and presented as syndecan-4 per cell relative to the mean of the sparse cells. The results are from five donors. Results are presented as mean with SEM denoted by vertical bars, and *p*-values < 0.05 were taken as a significant difference between the gene expression in sparse relative to dense cells using the Students paied *t*-test. * *p*<0.05, ** *p*<0.01, *** *p*<0.001. These results are presented as densitometric measurements in (D).

This striking effect of cell density on ^35^S-proteoglycan secretion was studied in further detail, using different experimental approaches. HUVEC have been reported to express several proteoglycans, including perlecan, biglycan, serglycin, syndecan-4 and versican [21, 34–38]. When comparing gene expression levels for these PGs it was evident that, irrespective of the proliferative status, perlecan was expressed at the highest level, followed by biglycan and serglycin, while the expression levels of versican and syndecan-4 were lower. This is shown for sparse cells in Fig 3A, the pattern was similar for dense cell cultures. Decorin as well as syndecan-1 and -2 were expressed at even lower levels [21].

The gene expression (Fig 3B) as well as secretion pattern (Fig 3C) of these most abundant endothelial PGs was compared in sparse and dense cultures. In contrast to all other proteoglycans investigated; only expression of serglycin mRNA increased with decreasing cell density. Perlecan, biglycan and versican mRNA expression was reduced with reducing cell density, while syndecan-4 mRNA was unaltered. The effect of cell density on proteoglycan secretion was also determined by Western blotting, immunoprecipitation or ELISA. The secretion levels corresponded to the gene expression levels, implying that serglycin increased in sparse cultures. Perlecan and versican secretion decreased; syndecan-4 was unaltered, while biglycan increased. Consequently, the expression and secretion of different proteoglycans are differently influenced by the proliferative status of these cells, with serglycin standing out as the major proteoglycan increasing in proliferating cell cultures.

**Fig 4:**
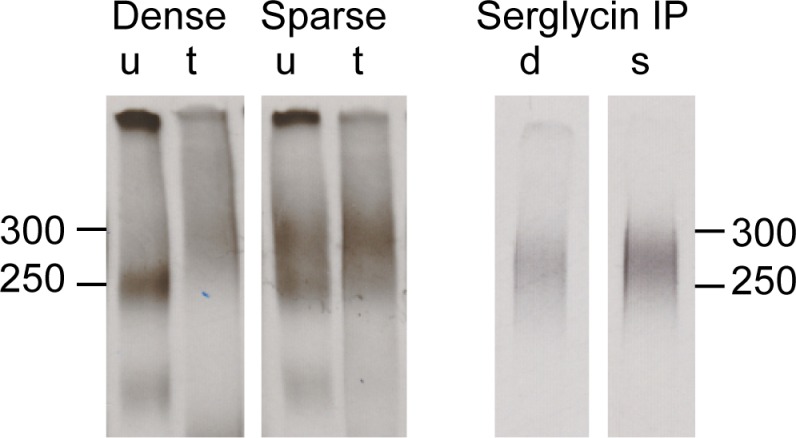
Characterization of the high molecular weight CS/DS-proteoglycan component. Left panel: Equal amounts (cpm) of untreated (u) and trypsin treated (t) ^35^S-PGs secreted from dense and sparse cells were separated by SDS-PAGE. Right panel: Immunoprecipitated (IP) ^35^S-labeled serglycin secreted from dense (d) and sparse (s) HUVEC. Sample size was adjusted to the protein content of the corresponding cell lysate. These results are from one representative culture of three donors analyzed. The migration positions of molecular mass markers are given in kDa.

The results from Fig 3 suggest that the observed increase in *de novo* ^35^S- proteoglycan secretion from Fig 2A is caused in part by increased secretion of serglycin. Furthermore, in proliferating cultures, there was an obvious increase in the 300 kDa material and a decrease in the 250 kDa material (Fig 2C). It has been shown that serglycin, unlike other proteoglycans, is resistant to degradation by the serine protease trypsin [39, 40]. This was exploited to further identify the components of this particular cell-density sensitive component. ^35^S- proteoglycans secreted from HUVEC were subjected to SDS-PAGE before and after trypsin treatment, as shown in Fig 4, left panel. The 300 kDa material which dominated in sparse cultures, was resistant to trypsin treatment while the 250 kDa material, dominating in the dense cultures, was degraded. Also, the molecular weight of the trypsin-resistant component corresponded to the molecular weight of ^35^S-serglycin identified by immunoprecipitation (Fig 4, right panel). In conclusion, these results identify the cell-density sensitive proteoglycan at approximately 300 kDa as serglycin.

### Serglycin localization

**Fig 5:**
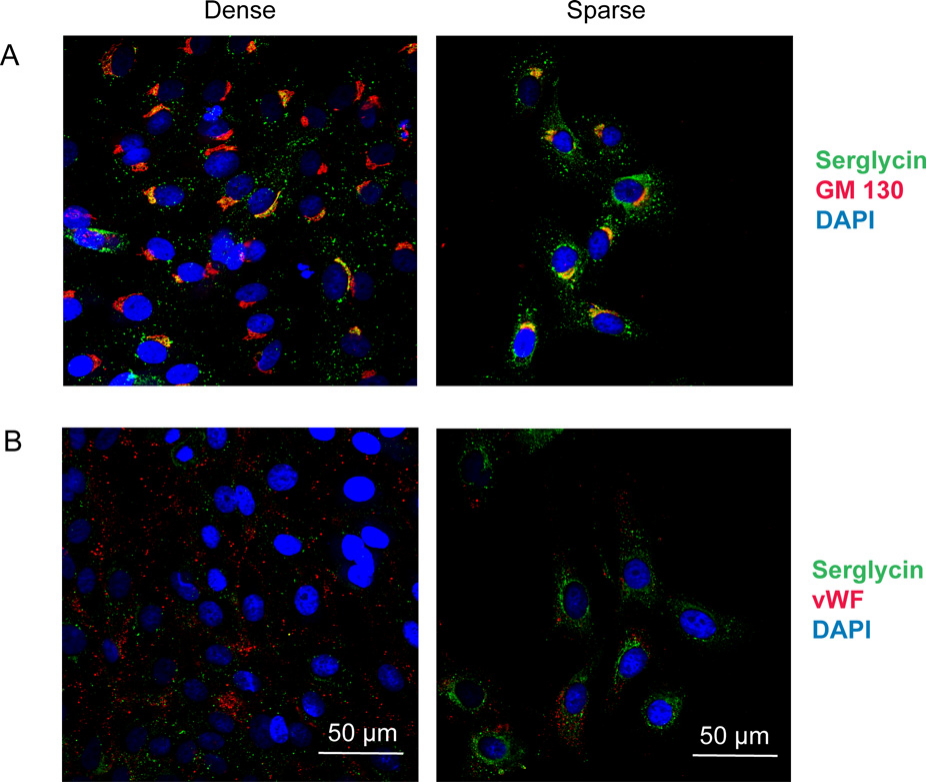
Intracellular distribution of serglycin, Golgi-marker and vWF in sparse and dense HUVEC. The left panel shows dense and the right panel shows sparse cultures of HUVEC. The cultures were fixed and stained for serglycin (green) and Golgi marker GM130 (A) in red or the endothelial marker vWF in red (B), with co-localization of the two visible as yellow. Blue color indicates DAPI nuclear staining. The pictures were acquired using a confocal microscope with 60 times magnification; scale bars shows 50 μm.

By immunocytochemistry, staining for both serglycin and the Golgi marker GM130, the intracellular distribution of serglycin differed between dense and sparse cultures (Fig 5A). In dense cultures limited staining of serglycin was seen in cytoplasmic vesicles and perinuclear regions and co-localized with the Golgi marker GM130. Vesicular staining increased in sparse cultures with an overt increase in Golgi staining. No co-localization with KDEL, a marker of endoplasmic reticulum, was observed in either sparse or dense cultures (results not shown). Staining for the endothelial specific marker vWF was evident in vesicles in both cultures, and merge of serglycin and vWF staining did not reveal any co-localization (Fig 5B), as previously has been shown for proliferating cells [21]. Hence, both the sparse and dense cultures express the established endothelial marker vWF.

### Serglycin in functional assays

Our results demonstrate an increased serglycin expression and secretion in proliferating HUVEC. Serglycin is a secretory proteoglycan with the ability to interact, through its glycosaminoglycan-chains, with several different partner molecules having the potential to regulate proliferation.

**Fig 6:**
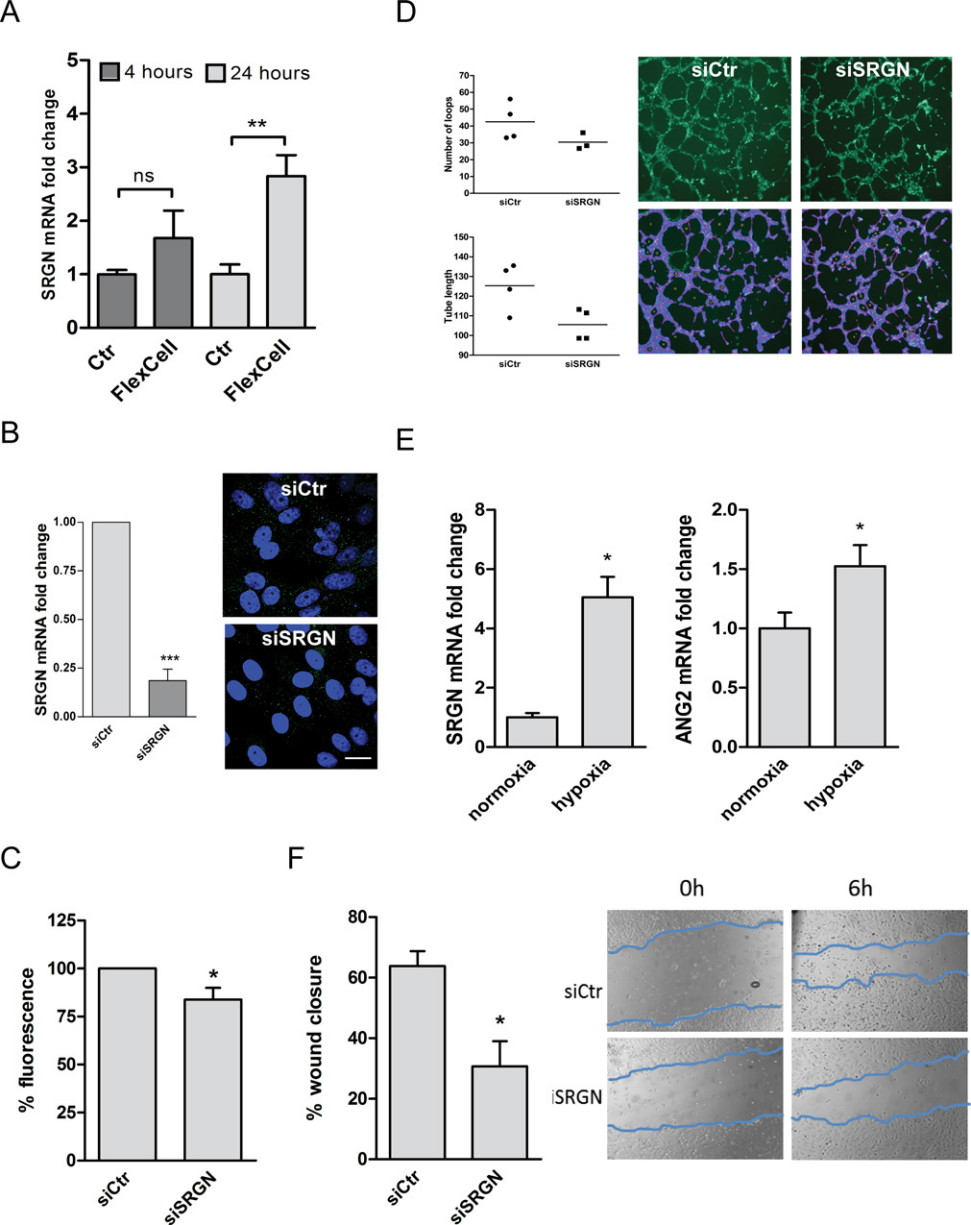
Serglycin in functional assays. (A) Proliferative capacity of serglycin knockdown cells (siSRGN) compared to scrambled controls (siCtr), n = 5. (B) HUVEC were subjected to 10% cyclic stretch for 4 and 24 hours respectively, and the response in serglycin expression was determined by qRT-PCR (FlexCell) and compared to controls (Ctr), n = 3–11. (C) Serglycin mRNA expression (qRT-PCR) and protein (ICC) in serglycin knockdown (siSRGN) in HUVEC was compared to scrambled controls (siCtr), n = 6. (D) Control and serglycin knockdown cells were subjected to an in vitro angiogenesis assay. Left panel show the quantification of tube length and loop numbers by Wimasis Image Analysis. The right panels show a representative picture of in vitro angiogenesis assay showing tube formation capacity on BME gel in siSRGN HUVEC and siCtr, n = 4. (E) Gene expression of SRGN (left) and ANG2 (right) in hypoxia compared to normoxia in triplicates from each of two cell donors. (F) Closure of scratch wound in control cells compared to siSRGN cells. Left panel show percent wound closure after 6 hours, and right panel show representative phase-contrast images of scratch wound at 0 and 6 hours after wounding, n = 3. Results are presented as mean with SEM denoted by vertical bars, and *p*-values < 0.05 were taken as a significant difference using the Students paired *t*-test. * *p*<0.05, ** *p*<0.01, *** *p*<0.001, ns: not significant.

To study the possible role of serglycin in the pulsatile shear stress induced in vivo by the hemodynamic forces, HUVEC were subjected to an experimental cyclic stretch assay (Flex Cell), and serglycin gene expression was determined after 4 and 24 hours. Serglycin mRNA increased 3-fold after 24 hours of stretch (Fig 6A). When knockdown of the serglycin gene was achieved in HUVEC by RNAi silencing (Fig 6B) their proliferative capacity was significantly reduced (Fig 6C).

*In vivo*, endothelial cells are quiescent for years, but when tissues are deprived of oxygen or nutrients, they sprout to vascularize tissues. To investigate the functions of serglycin in angiogenesis, control and serglycin knockdown cells were subjected to an in vitro angiogenesis assay. The results showed a small (non-significant) reduction of both number of loops and mean tube length in the absence of serglycin (Fig 6D). In a hypoxic environment, SRGN expression increased 5-fold after 24 hours (Fig 6E). Hypoxia-induced angiogenesis is regulated by multiple molecular effectors including the growth factor Angiopoietin-2 (Ang2) [41]. Ang2 increased also in our system (Fig 6E), suggesting involvement of serglycin as well as Ang2 in angiogenesis. Another experimental approach to study the role of serglycin in regulation of proliferation as well as migration is to use an *in vitro* scratch wound assay. This approach showed a reduced closure of the scratch wound in the serglycin knockdown cells compared to the scrambled controls (Fig 6F). Serglycin stainings revealed increased perinuclear expression in cells in the wound edge (S1 Fig) resembling the pattern observed in the sparse cultures.

### Serglycin in endothelial IL-1β activation

**Fig 7:**
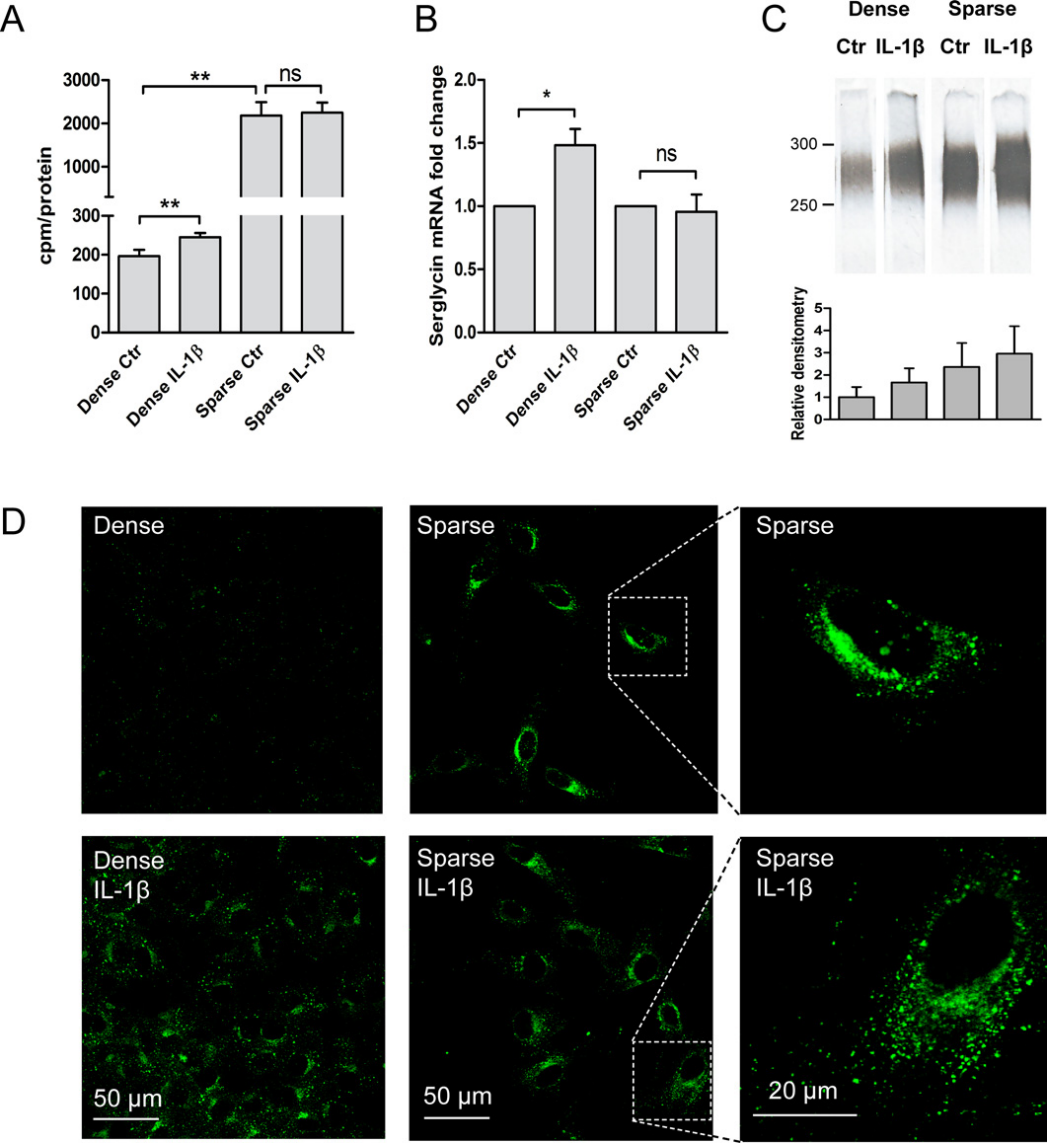
Effect of IL-1β on sparse compared to dense cell cultures. HUVEC were cultured in high (dense) or low (sparse) cell density for 24 hours with or without IL-1β. (A) HUVEC from 6 donors were metabolically labeled with ^35^S-sulfate for 24 hours and the ^35^S-proteoglycan secretion was determined by scintillation counting and related to protein content. (B) SRGN mRNA expression in response to IL-1β activation for dense and sparse cultures (n = 6). (C) Serglycin secretion was determined by immunoprecipitation of ^35^S-serglycin from conditioned medium. These results are from one representative culture of three donors analyzed (upper panel) and mean densitometric measurements of results from all donors (lower panel). The migration positions of molecular mass markers are given in kDa. (D) Confluent (dense) and sparse HUVEC cultures were cultured on chamber slides, fixed and stained for serglycin, and pictures were acquired using a Olympus Fluo View FV1000 confocal microscope with 60x magnification. The intracellular localization and expression of serglycin after IL-1β stimulation is compared to unstimulated control cells in both dense (left panel) and sparse (middle panel) cultures. The right panel show a 3.7 times magnification of the indicated selected areas and the scale bars indicate 20 and 50 μm respectively. Results are presented as mean with SEM and differences with p<0.05 was regarded as statistically significant using the Students paired *t*-test. * *p*<0.05, ** *p*<0.01, ns: not significant.

In the processes of wound healing, angiogenesis and tissue repair, inflammatory responses are of central importance. IL-1β is an important factor in the pathogenesis of type 2 diabetes [10]. Previous studies in confluent HUVEC demonstrated that the proinflammatory mediator IL-1β has a stronger influence on heparan sulfate proteoglycans than several other classical inflammatory cytokines [20], and caused a 1.5 fold increase in ^35^S-proteoglycan secretion [42]. In both dense and sparse HUVEC, proliferation rate and cytotoxicity was not influenced by IL-1β (S2 Fig). However, IL-1β increased ^35^S-proteoglycan secretion in dense cultures only (Fig 7A). The increase in ^35^S-proteoglycan secretion in dense cultures, but not in sparse was reflected in the serglycin mRNA level (Fig 7B). Furthermore, immunoprecipitation showed an increased secretion in both sparse and dense cells, but more in dense cell cultures (Fig 7C). The secretion of serglycin is lower in unstimulated dense than in sparse cultures, as previously observed in Figs 3 and 4. Supporting this finding, serglycin-positive vesicles increased after IL-1β stimulation both in sparse and in dense cultures as demonstrated by immunofluorescence (Fig 7D).

### Serglycin and partner molecules

**Fig 8:**
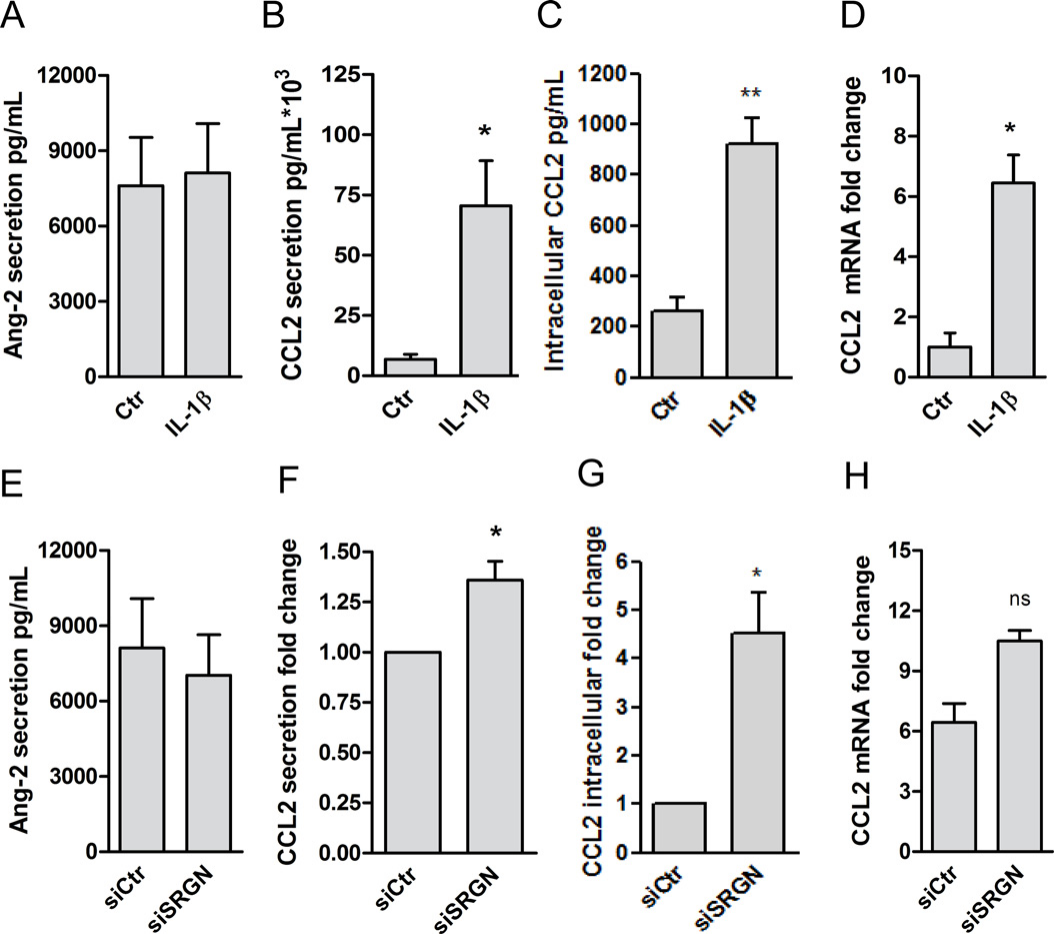
CCL2 and Ang2 response to inflammation and to serglycin knockdown. The secretion of Ang2 (A, n = 6) in control cells was compared to IL-1β stimulated cells. Also, secretion (B, n = 6), intracellular levels (C, n = 4) and gene expression (D, n = 4) of CCL2 in control cells was compared to IL-1β stimulated cells. Response in Ang2 secretion (E, n = 6) and CCL2 secretion (F, n = 6), intracellular levels (G, n = 4) and gene expression (H, n = 4) in serglycin knockdown cells (siSRGN) were compared to control cells (siCtr). Results are presented as mean with SEM and differences with p<0.05 was regarded as statistically significant using the Students paired *t*-test. * *p*<0.05, ns: not significant.

We hypothesized that serglycin may exert its regulatory functions trough interactions with transport, protection and presentation of chemokines, cytokines and growth factors. Endothelial cell stimulation via IL-1 signaling results in an increased release of growth factors including Ang2, VEGF and PDGFA as well as chemokines including CXCL1 (GROα), CCL2 (MCP1) and CCL3 (MIP1α). Ang2, VEGF and PDGF are all angiogenic growth factors expressed by HUVEC and dependent on heparan sulfate for its biological effects [43, 44]. In our hands however, secretion levels of VEGF and PDGF-B in HUVEC were undetectable and not induced by IL-1β (results not shown). In contrast, the secretion of Ang2 was high (Fig 8A), but not significantly increased by IL-1β. Serglycin has been shown to colocalize with CXCL1 in type 2 granules, and we have shown that CXCL1 secretion is dependent on serglycin [21].

CCL2 is a pro-inflammatory and angiogenic chemokine [45], also found in type 2 granules of HUVEC [46]. We here confirm the results from previous studies showing that CCL2 is highly expressed by HUVEC, demonstrating a 3,5 fold increase in intracellular CCL2 (Fig 8C), a 16-fold increase in CCL2 secretion in IL-1β-stimulated cells (Fig 8B) and a 6.4-fold increase in mRNA expression (Fig 8D). In macrophages CCL3 is associated with serglycin [47], However, in HUVEC we could not detect secreted CCL3 (results not shown).

In light of these results, we explored the possible role of serglycin in the secretion of Ang2 and CCL2 upon IL-1β stimulation. For this purpose, serglycin gene expression was reduced using RNAi silencing of the SRGN gene transcript. From Fig 8E it is evident that this did not affect secretion of Ang2. However, both intracellular and secreted CCL2 was increased in serglycin knockdown cells (Fig 8F and 8G). This was accompanied by a small, but non-significant increase in CCL2 gene expression (Fig 8H).

**Fig 9:**
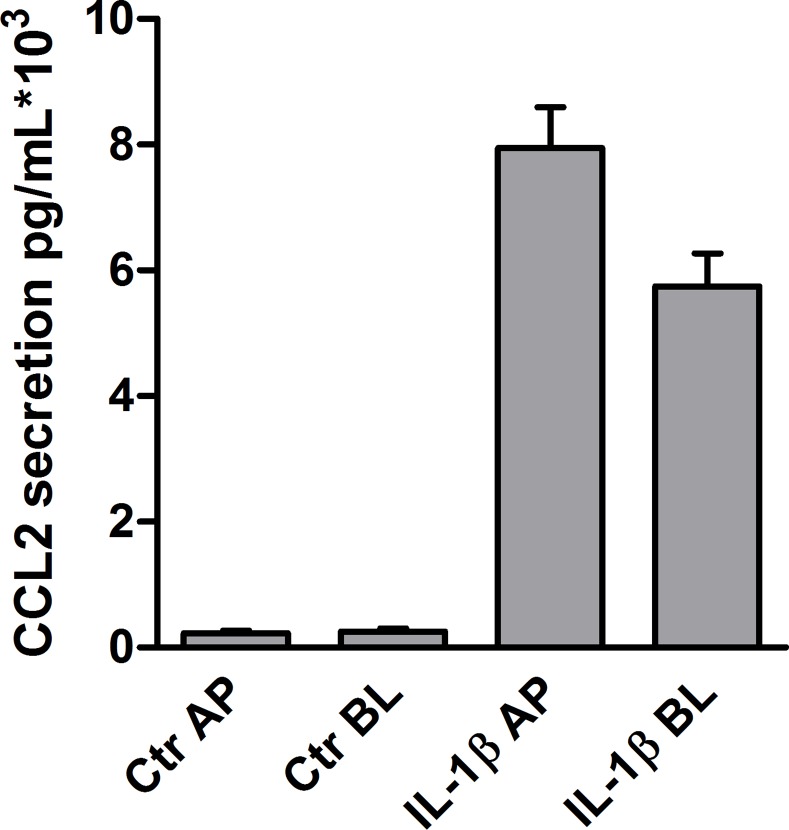
CCL2 secretion from polarized cells. HUVEC from three different donors were polarized on semipermeable filters. Confluent monolayers were untreated (Ctr) or incubated with IL-1β for 24 hours. CCL2 content in apical (AP) and basolateral (BL) conditioned media was compared.

To further explore the role of serglycin in CCL2 storage and secretion, HUVEC were cultured on semipermeable filters, allowing the cells to polarize. This system makes it possible to study secretion to the apical and basolateral side of confluent endothelial monolayers. We have earlier shown that serglycin is secreted predominately to the apical side of polarized HUVEC cells. Interestingly, we show here that the increase in CCL2 secretion upon IL-1β stimulation was also predominately to the apical side of the cells (Fig 9). In serglycin knockdown cells, secretion to the apical side increased 12,4 fold (± 6.332) compared to a modest 1.699 fold (± 2.498) decrease to the basolateral side. This result supports a possible role for serglycin in the secretion of the chemokine CCL2.

**Fig 10:**
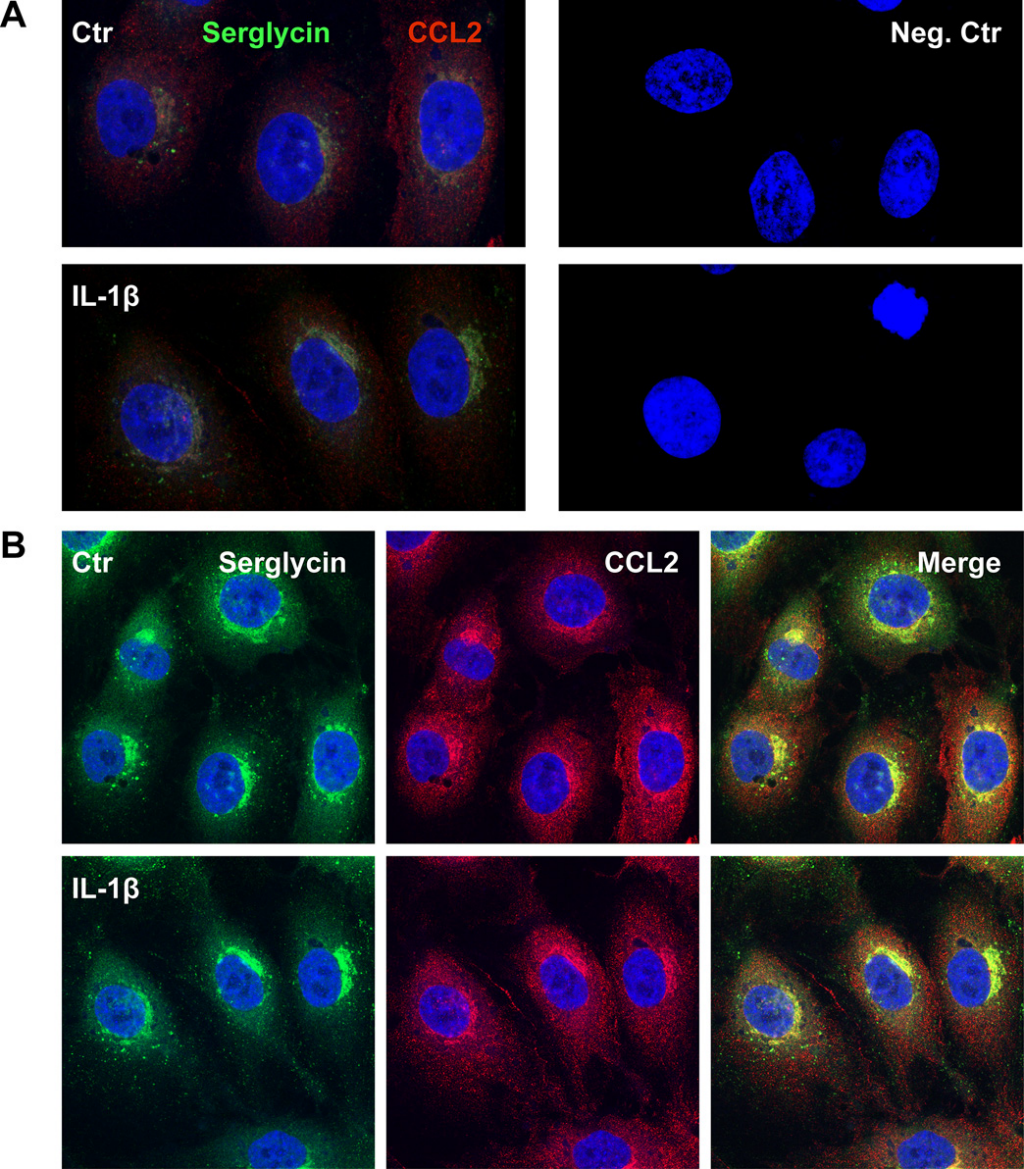
Serglycin and CCL2 distribution in unstimulated and stimulated cells. HUVEC were cultured on chamber slides for 24 hours without (Ctr) or with IL-1β. The cells were fixed and stained, and pictures were acquired using a Zeiss LSM 700 confocal laser scanning microscope at 62x magnification. These are representative results from cells isolated from three different donors. (A) The left panel show cells stained for serglycin in red and CCL2 in green. The right panel show the negative control stained with secondary antibodies only. These pictures are Z-stacks showing the distribution of the target proteins throughout the cells. (B) The panels show untreated (top) and IL-1β stimulated (bottom) HUVEC cultures, fixed and immunostained for serglycin (green) and CCL2 (red). The left and middle panels show serglycin and CCL2 staining, respectively. The right panel shows the overlay (merge) of serglycin and CCL2. These pictures are the view from the middle of the cells, were both target proteins are expressed equally. The scale bar indicates 10 μm.

Finally, cells were fixed and stained for serglycin and CCL2 both in unstimulated (Ctr) and IL-1β stimulated cells and blinded confocal microscopy analysis was performed (Fig 10). Fig 10A show Z-stacks illustrating the distribution of the target proteins throughout the cells, while Fig 10B show one focal plane in the middle of the cells, where both proteins are equally expressed. Serglycin was observed in perinuclear regions and in vesicles of different sizes. In stimulated cells, serglycin perinuclear staining was increased, indicating increased synthesis. The vesicular staining was increased upon IL-1β stimulation, and serglycin positive vesicles were observed in higher distance from the nucleus, suggesting an increased secretion of serglycin in these cells. CCL2 on the other hand was observed in perinuclear regions and also in small vesicles throughout the cytoplasm. In unstimulated cells, CCL2 staining was visible also at the cell surface, mostly in areas with cell-cell contact. In IL-1β stimulated cells, this cell surface staining was reduced, indicating a secretion of preformed CCL2 from these areas. Perinuclear staining increased, suggesting increased synthesis of CCL2. Co-localization of serglycin and CCL2 is visible as yellow. We observed overt co-localization of serglycin and CCL2 in perinuclear regions. Co-staining was also present in a portion of the small vesicles, and in stimulated cells increased costaining was observed in the cytosol.

## Discussion

Proteoglycans are important components of the matrix and play important roles in signaling related to cellular event such as inflammatory response and proliferation. In this study we observe a 9-fold increase in ^35^S-proteoglycan secretion in proliferating endothelial cells compared to quiescent cultures, and we identify serglycin as an important component of the cell-density sensitive proteoglycan population. In line with this, we also provide evidence that gene expression, secretion and intracellular distribution of serglycin is strikingly different between sparse and dense cultures. However, our knowledge of the possible roles and mechanisms of serglycin expressed by endothelial cells is limited. Most previous studies have focused on serglycin in hematopoietic cells [22], although serglycin is also expressed by other cells including smooth muscle cells [48], cancer cells [25, 26], endothelial cells [35] and adipose tissue [49].

In human endothelial cells we compared the expression of serglycin to several other proteoglycans, both basement membrane proteoglycan perlecan, the small leucine rich repeat proteoglycan biglycan, the large connective tissue proteoglycan versican, and the cell surface proteoglycan syndecan-4. Our gene-expression data pointed to perlecan as the major proteoglycan in these primary endothelial cells, followed by serglycin and biglycan, whereas the other proteoglycans were expressed at lower levels. Interestingly, expression and secretion of serglycin increased in proliferative compared to quiescent cultures. This was in contrast to all the other proteoglycans examined, which were reduced or unaffected. This observation is in line with an early study on monocytes, which mainly express serglycin, demonstrating high proteoglycan expression in low density cell cultures and lowered expression in high density cultures [50], and a study in endothelial cells demonstrating cell density-dependent regulation of proteoglycan synthesis [51]. The increase in perlecan and versican in quiescence compared to actively proliferating cells could reflect the need for proteoglycans in the construction of endothelial basement membrane and extracellular matrix in dense cultures, compared to actively proliferating cells.

Our data may suggest other possible functions for serglycin in endothelium related to the proliferative status of the cells. It is therefore of interest that serglycin is involved in the regulation of apoptosis in mast cells [52] and that lymphoid tissues are enlarged in aging serglycin ^-^/^-^ mice, which may be due to serglycin affecting proliferation, apoptosis or both [53]. Furthermore, high levels of serglycin were demonstrated in nasopharyngeal carcinoma cells, with higher levels in clones with increased metastatic potential and also, with higher motility [26]. Recently, serglycin expression was related to cancer aggressivity both in tumor and stroma [54, 55].

Wound healing occurs as a cellular response to injury and involves activation of endothelial cells. The endothelium is especially important in the inflammatory, proliferative and angiogenic phases of wound healing. Many growth factors and cytokines released by endothelial cells are needed to coordinate and maintain the healing, and endothelial proliferation and angiogenesis is vital. In diabetes we see impaired wound healing, leading to complications such as foot ulcers. Furthermore, in tumors, an abnormal endothelium contributes to tumor growth and metastasis [7]. We show reduced proliferative and wound-healing capacity in endothelial cells after serglycin knockdown. Accordingly, the results presented here implicate serglycin in endothelial dysfunction relevant for both diabetic complications and cancer.

A reduction in normal tissue oxygen triggers compensatory responses such as angiogenesis, resulting in improved tissue oxygenation. Although angiogenesis was not significantly influenced in our experiments; serglycin increased in response to hypoxia. Early findings demonstrated a reduced endothelial proteoglycan secretion in hypoxia, assessed by ^35^S-sulfate metabolic labelling [56]. In macrophages however, a more recent study demonstrated an increase in the proteoglycans versican and perlecan, mediated trough the HIF-pathway [57]. Furthermore, the novel hypoxia-sensitive chondroitin sulfate proteoglycan CSPG4, important in proliferation, is also increased in hypoxia [58].

The effect of serglycin expression on proliferation has been studied in tumor models. Purushothaman and Toole found that serglycin knockdown in myeloma cells reduced tumor growth [23]. A recent study found enhanced tumor growth and proliferation in serglycin deficient RIP1-Tag2 mice [59]. This is in contrast to the results presented here. Notably, serglycin might have different functions depending on the tissue or cell type in question.

Not only proliferation, but also the migratory capacity will affect the outcome of the scratch wound assay. It is of interest that serglycin is shown to affect the migratory capacity of cancer cells [25], suggesting an effect of serglycin on migration also in endothelial cells. However, preliminary data from our laboratory suggest that migration is not decreased in the serglycin knockdown. We observed that quiescent HUVEC increased the serglycin expression more than proliferating cells after IL-1β stimulation. Proliferating cells have a high expression of serglycin, and inflammatory stimuli are not able to increase this further, in contrast to resting cells. Together, these results support a role for serglycin not limited to proliferative processes, but also in inflammatory reactions. It can be hypothesized that serglycin is more involved in processes related to proliferation in sparse endothelium, while more devoted to inflammatory reactions in quiescent cells. The mechanisms by which serglycin is involved in both inflammatory reactions and regulation of proliferation has only been studied to a limited extent [22]. However, in a recent study it was demonstrated that UV irradiated skin of human volunteers had accumulations of dermal and endothelial serglycin [60], showing that activated endothelial cells express serglycin *in vivo*. This *in vivo* finding is in accordance with data presented here and supports the notion that serglycin in endothelial cells can be involved in both endothelial inflammatory reactions and regulation of apoptosis and proliferation.

Serglycin has the ability to interact with partner molecules through the glycosaminoglycan side-chains [61]. The importance of serglycin for the storage of proteases and histamine in granules in mast cells [62], for granzyme B storage in cytolytic T-cells [63], for chemokine and platelet derived growth factor storage in platelets [64] and for TNFα secretion in macrophages [65] has been clearly documented. In both storage granules and secretory vesicles serglycin interacts with several cytokines and growth factors. Several of these are involved in angiogenesis and wound repair [66, 67]. We have previously established that serglycin is a major proteoglycan in primary human endothelial cells, involved in the storage and secretion of the chemokine CXCL1, which was reduced in the absence of serglycin [21, 42].

To understand more of the mechanisms behind the functions of serglycin in proliferation and inflammation, we knocked down serglycin expression. In serglycin knockdown cells, the CCL2 secretion in response to IL-1β activation was significantly increased. Furthermore, we found that both serglycin and CCL2 was secreted predominately to the apical side of polarized endothelial cells. Finally, serglycin and CCL2 co-localized not only in perinuclear areas, but also in some of the vesicles. Together these data provide evidence for a role of serglycin also in the regulation of secretion CCL2. CCL2 is a pro-inflammatory chemokine involved in cancer metastasis by promoting monocyte attraction and angiogenesis [68]. Notably, the effect of serglycin knockdown was opposite for CCL2 and CXCL1, pointing to different mechanisms for serglycin regulation of these two chemokines. Possibly, serglycin could be important for the vesicular storage of CCL2, while more involved in the secretion of CXCL1. These results are also comparable to the increased secretion of TNFα observed in macrophages derived from SRGN-/- mice [65]. A role for serglycin in intracellular storage or in lysosomal targeting, are possible scenarios. Alternatively, with its ability to bind proteases, serglycin could have extracellular functions affecting the transport and protection of secreted chemokines. In contrast to these two chemokines, we show that Ang2 secretion is not dependent on serglycin, indicating differences in regulation of secretion of these angiogenic factors. Endothelial cells express CCR2, the receptor for CCL2. IL-1β stimulation and scratch wound injury increase this expression [69]. HUVEC also respond to CCL2 by increased angiogenesis [70], providing a possible autocrine mechanism for regulation of proliferation. It is possible that serglycin participates both in secretion of CCL2 and presentation of CCL2 to CCR2, in line with what has been shown for delivery of serglycin in multimeric complexes with perforin and granzyme B to target cells [71].

**Fig 11:**
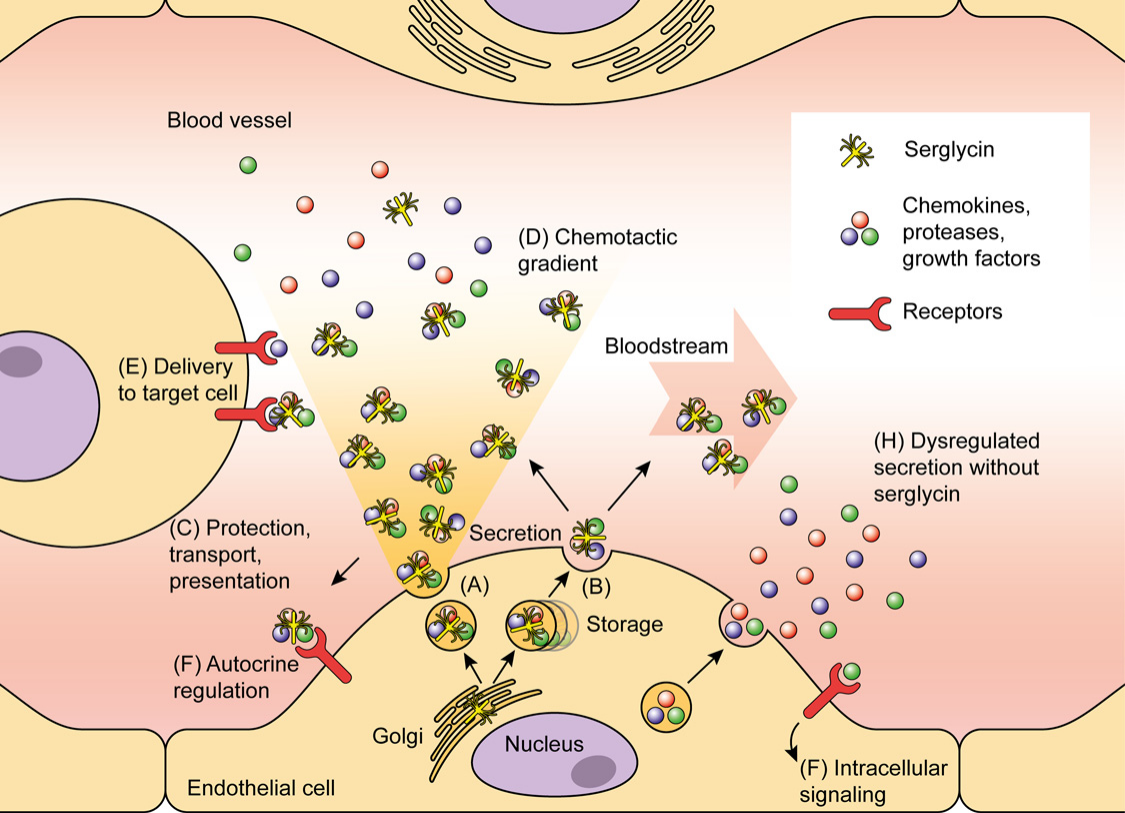
Possible mechanisms for the role of serglycin in regulation of proliferation. In endothelium, serglycin is subject to constitutive (A) and regulated (B) secretion. Constitutive secretion occurs mainly to the apical side, facing the bloodstream, but also to the basolateral side. Vesicles for regulated secretion might be stored intracellularly and serglycin is secreted mainly to the apical side upon stimulation. Through the glycosaminoglycan (GAG)-chains, serglycin has the ability to bind partner molecules including chemokines, growth factors and proteases, and thus secreted serglycin can offer protection, transport and presentation of these molecules (C). Hence, serglycin can contribute to the formation of chemotactic gradients [72] of both bound and dissociated effector molecules (D). This can have autocrine effects (E) as well as effects on target cells (F). CCL2 is recognized as an angiogenic chemokine [45], and in serglycin knockdown cells we observe an increase in CCL2 secretion. Lack of serglycin has several possible outcomes. It could result in a lack of gradient formation, as well as reduced transport and presentation affecting target cells and autocrine signaling. Further, absence of serglycin could affect storage and secretion, resulting in dysregulated secretion exemplified by the increased levels of CCL2 observed here (G). This can have consequences for the autocrine effect of CCL2 on endothelium, suggesting that serglycin is necessary for chemokine presentation to its receptor.

A tentative scheme for possible mechanisms of the effects of serglycin in regulation of proliferation is provided in Fig 11. In this figure we suggest that serglycin can be important for endothelial inflammatory response, proliferation and angiogenesis such as in wound healing; through regulation of secretion, transport, protection and presentation of partner molecules including CCL2.

Together, our findings suggest that serglycin expression and localization is related to the proliferative status of the cells. This is most probably achieved through interactions with and regulation of partner molecules. The cell density dependent expression of serglycin can reflect an increased demand for interactions with pro-angiogenic factors. This knowledge is important for further understanding of functions and mechanisms of serglycin in endothelial cells in tumor biology and development of diabetic complications.

## Supporting Information

S1 Fig Intracellular distribution of serglycin and VE-cadherin staining in wound-assay. HUVEC cultures with wound (right panel) or with no wound (left panel) were fixed and stained for serglycin (green) and VE-Cadherin (red). Blue color indicates DAPI nuclear staining. VE-Cadherin is expressed in endothelial cell junctions. In wounded areas with reduced VE-Cadherin expression, serglycin perinuclear expression is increased. The pictures were acquired using a confocal microscope with 60 times magnification.
(TIF)

S2 Fig Effect of IL-1β on cell proliferation and cytotoxicity in sparse and dense cells. (A) Cytotoxicity of IL-1β on HUVEC from 2 donors was determined as a function of time using the LDH-assay in sparse (left panel) and dense (right panel) cell cultures. The results are presented as % mortality of maximum. (B) The proliferation rate was assessed by applying the MTS-assay on cell cultures of varying densities. This assay was performed with cells from 4 donors for all cell densities for control cells (Ctr) and 3 donors for IL-1β stimulated cells (IL-1β) at 2 500, 10 000 and 50 000 cells per well, and from 1 donor for 5 000 and 25 000 cells per well of 0.32 cm^2^. Each point shows the mean absorbance per cell, reflecting the cell proliferation. All means are shown with SEM denoted by vertical bars.
(TIF)

## Abbreviations

cABC: chondroitinase ABC
CS: chondroitin sulfate
DS: dermatan sulfate
HUVEC: human umbilical vein endothelial cells
vWF: von Willebrand Factor
