# Supplementary figures and images for: Serglycin in Quiescent and Proliferating Primary Endothelial Cells
Source: PLoS One. 2015 Dec 22;10(12):e0145584. doi: 10.1371/journal.pone.0145584 (PMC4687888; doi:10.1371/journal.pone.0145584)

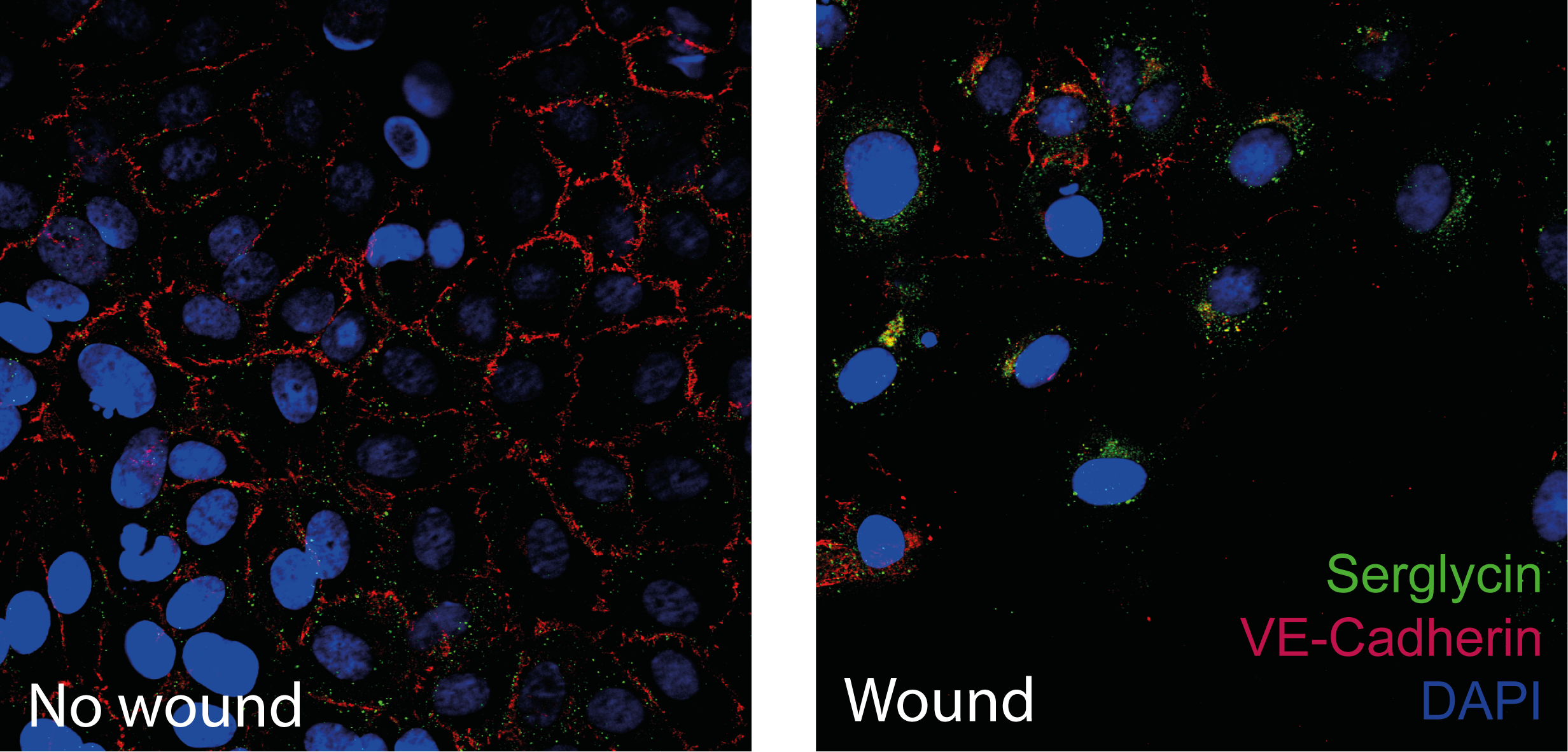

Supplement: S1 Fig — HUVEC cultures with wound (right panel) or with no wound (left panel) were fixed and stained for serglycin (green) and VE-Cadherin (red). Blue color indicates DAPI nuclear staining. VE-Cadherin is expressed in endothelial cell junctions. In wounded areas with reduced VE-Cadherin expression, serglycin perinuclear expression is increased. The pictures were acquired using a confocal microscope with 60 times magnification. (TIF) [file pone.0145584.s001.tif]

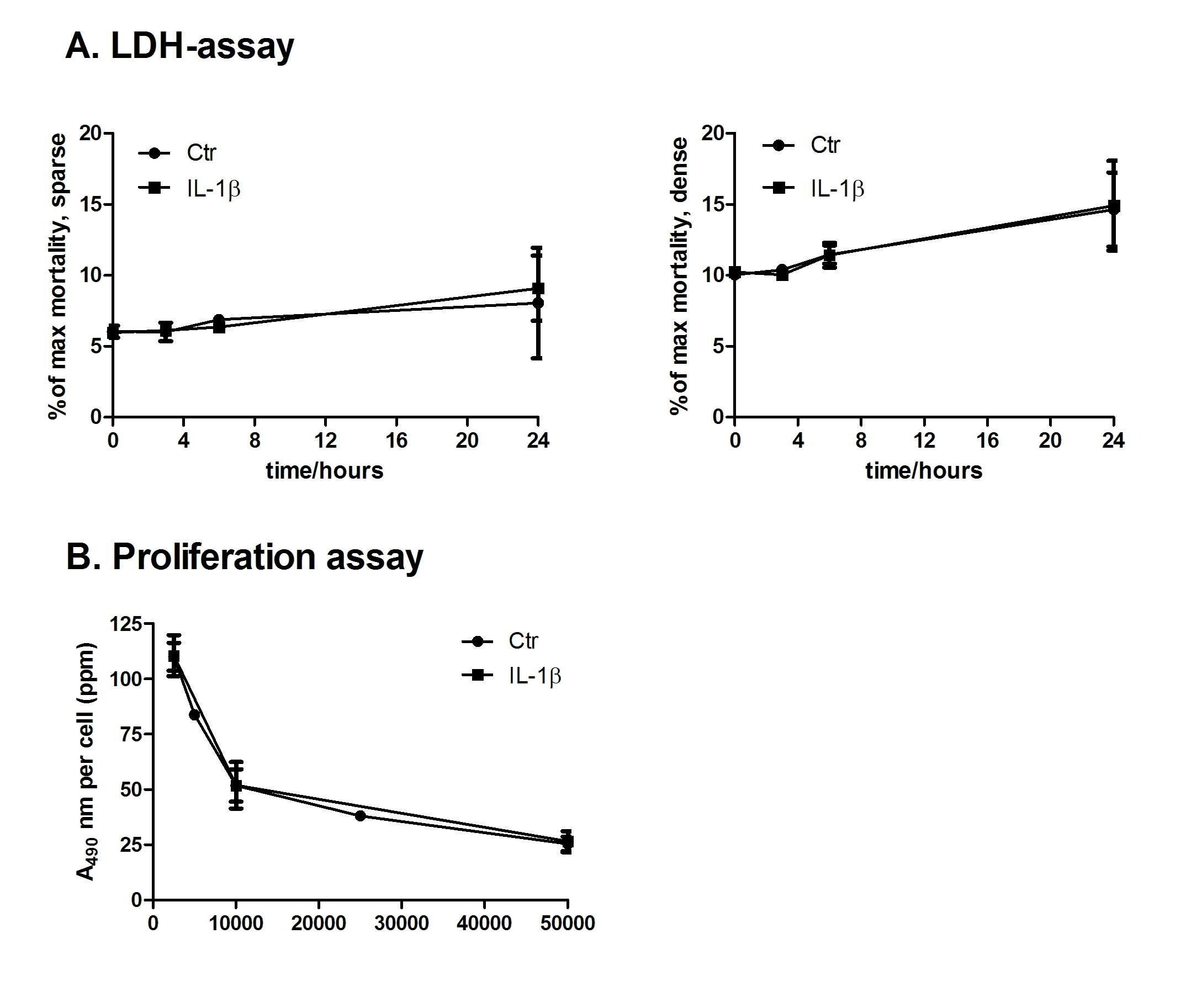

Supplement: S2 Fig — (A) Cytotoxicity of IL-1β on HUVEC from 2 donors was determined as a function of time using the LDH-assay in sparse (left panel) and dense (right panel) cell cultures. The results are presented as % mortality of maximum. (B) The proliferation rate was assessed by applying the MTS-assay on cell cultures of varying densities. This assay was performed with cells from 4 donors for all cell densities for control cells (Ctr) and 3 donors for IL-1β stimulated cells (IL-1β) at 2 500, 10 000 and 50 000 cells per well, and from 1 donor for 5 000 and 25 000 cells per well of 0.32 cm2. Each point shows the mean absorbance per cell, reflecting the cell proliferation. All means are shown with SEM denoted by vertical bars. (TIF) [file pone.0145584.s002.tif]
